# Laser interferometry analysis of ciprofloxacin and ampicillin diffusion from liposomal solutions to water phase

**DOI:** 10.1007/s00249-013-0904-2

**Published:** 2013-04-21

**Authors:** Sławomir Wąsik, Michał Arabski, Zuzanna Drulis-Kawa, Jerzy Gubernator

**Affiliations:** 1 Department of Molecular Physics, Institute of Physics, Jan Kochanowski University, Świętokrzyska 15, 25-406 Kielce, Poland; 2 Department of Microbiology, Institute of Biology, Jan Kochanowski University, Świętokrzyska 15, 25-406 Kielce, Poland; 3 Institute of Genetics and Microbiology, University of Wrocław, Przybyszewskiego 63/77, 51-148 Wrocław, Poland; 4 Department of Lipids and Liposomes, Faculty of Biotechnology, University of Wrocław, Przybyszewskiego 63/77, 51-148 Wrocław, Poland; 5 Academic Centre for Biotechnology of Supramolecular Lipid Aggregates, Przybyszewskiego 63/77, 51-148 Wrocław, Poland

**Keywords:** Laser interferometry, Diffusion, Liposomes, Ciprofloxacin, Ampicillin

## Abstract

The paper presents experimental investigations of diffusion of antibiotics (ciprofloxacin or ampicillin) into the water phase from mixtures of neutral or negatively charged liposomes, and antibiotic–liposome interactions. Using the laser interferometry technique, the amounts and fluxes of released antibiotics, concentration field evolution, and the velocity of the concentration boundary layer’s “growth” were determined. To avoid the limitations of membranes, a measurement system without the artificial boundary of phases with a free water–solution interface has been proposed. It was found that the diffusion of anionic and neutral liposomes into the water phase was insignificant and mainly the diffusion of antibiotics was measured. Differences in the diffusion kinetics of ciprofloxacin and ampicillin from liposomal solutions to the water phase were observed. Ampicillin diffused more efficiently than ciprofloxacin regardless of the liposomal solution type. Moreover, the amount of ampicillin and ciprofloxacin released from the anionic liposomal phase was higher than that from the neutral one. Our results confirm that ciprofloxacin at neutral pH shows little tendency to bind neutral liposomes. Additionally, it was also observed that ciprofloxacin disrupts negatively charged liposomes as a final effect of antibiotic–lipid interactions.

## Introduction

Intensive research is being carried out on liposomal formulations of antibiotics to improve their pharmacokinetic properties and antimicrobial activity (Bakker-Woudenberg 1995; Pinto-Alphandary et al. 2000). Liposomes as antibiotic carriers have a significant effect on drug distribution improvement and drug toxicity decrease. The pharmacokinetics and antibacterial activity of liposomal antibiotics can be modified by several means. The different properties of liposomes depend on their size and lipid composition (charge and fluidity) (Sachetelli et al. 1999; Schiffelers et al. 2001; Webb et al. 1998). The encapsulation also affects drug pharmacodynamics; thus, precise analysis determining biochemical interactions between antibiotic and lipid vesicle membrane has to be carried out. Pharmacodynamic analysis describing diffusion rate and drug–lipid interactions may be carried out by application of the interferometric method.

A modification of the laser interferometric technique by immobilizing the tested molecules in agarose gel and measuring the amount of released substances as used by our team allowed us to determine the interactions within partially insoluble mixtures such as lipopolysaccharide (LPS)–colistin (Arabski et al. 2007), LPS–chitosan (Arabski et al. 2009a), and LPS–saponin (Arabski et al. 2009b). Moreover, this technique has been successfully applied in quantitative analysis of ethanol, glucose, and sucrose transport through nucleopore and cellulose membranes (Dworecki and Wąsik 1997; Dworecki et al. 2000, 2003, 2005a, b, 2006; Ślęzak et al. 2005; Wąsik et al. 2010). In the above laser interferometry applications, membrane or agarose gel was used as the artificial boundary of phases. However, the application of a small-pore membrane may make the substance transport and proper interpretation of the diffusion process difficult. Because of these limitations, a measurement system without the artificial boundary of phases has been proposed. The system contains a horizontally located nucleopore membrane with large pore diameter to avoid the hydrodynamic disturbances during the experimental model preparation only. This system with hydrophilic/hydrophobic solutions and large-pore membrane allows one to obtain a free interface and makes it possible to perform direct investigations of substance transport. The novel application of the laser interferometry method might be useful for quantitative analysis of antibiotic diffusion from a liposomal mixture to the water phase.

In our previous study, the kinetics of diffusion of empty cationic liposomes through nucleopore and cellulose membranes was analyzed (Arabski et al. 2012). Moreover, the experiments revealed that the amount of neutral or anionic liposomes transported through a nucleopore membrane to the water phase is statistically not significant (results not shown).

The present study was designed to determine the interaction between free drug and liposomal vesicles affecting diffusion efficacy. The experiments focused on: (a) quantitative analysis of antibiotic (ciprofloxacin or ampicillin) diffusion to the water phase from mixtures of neutral or negatively charged liposomes, and (b) antibiotic–liposome interactions. The aqueous solution of lipid formulation (first component of experimental configuration) and antibiotic (second component) was applied. This study has shown that modified laser interferometry can be an accurate and convenient method for evaluation of antibiotic diffusion rate on the edge of hydrophobic (liposomes) and hydrophilic (water) phases and to investigate lipid–antibiotic interactions. These interactions between drugs and lipids may be critical for the pharmacokinetic and dynamic activity of antibiotics.

## Materials and methods

### Membrane properties

Polymeric nuclear track membrane (nucleopore) with pore diameter 0.2 μm was purchased from the Joint Institute for Nuclear Research in Dubna, Russia.

### Chemicals

Phosphatidylglycerol (PG) and phosphatidylcholine (PC) were purchased from Northern Lipids Inc. (Vancouver, BC, Canada). Cholesterol (Chol) was obtained from E. Merck (Darmstadt, Germany). High-performance liquid chromatography (HPLC) solvents were supplied by J. T. Baker (Deventer, The Netherlands). Ampicillin and ciprofloxacin were obtained from Polfa Tarchomin (Warsaw, Poland) and Krka (Nove Mesto, Slovenia), respectively.

### Liposome preparation

A lipid formulation of negatively charged vesicles PC/CHOL/PG 3/4/3 (−28.3 ± 2.4 mV) and neutral vesicles PC/CHOL 6/4 (−3.1 ± 1.5 mV) was prepared for the study. Appropriate amounts of lipids dissolved in chloroform (10 mg/ml) were mixed in a 100-ml round-bottom flask. By evaporating the organic solvent at 40 °C, a thin film of dry lipid was formed on the inner wall of the flask. Residual solvent was removed under a high vacuum applied for at least 1 h. The dry lipid films (with 30 mg of total lipid) were hydrated by adding 1 ml of phosphate-buffered solution (PBS) (pH 7.2–7.4). Hydration was performed at a temperature maintained above the phase-transition temperature of the main liposome lipid (20 °C for PC) and was facilitated by adding two 5-mm glass beads and vortexing the liposomal suspension–multilamellar vesicle (MLV) formation. Unilamellar liposomes (ULVs) were prepared by extrusion (10×) through two stacked polycarbonate filters of 100-nm pore size (Nucleopore, Whatman) at 20 °C and at 50 °C on a Thermobarrel Extruder (Lipex Biomembranes, Vancouver, BC, Canada). The mean vesicle size was between 107 and 152 nm. Opalescent liposome fractions were collected, and lipid concentrations were then determined. Liposome size (multimodal analysis, volume weighted) and zeta potential were routinely determined on a Zetasizer 5000 (Malvern Instruments Ltd., Malvern, UK). Lipid concentration in the range of 15 mg/ml for both formulations was determined colorimetrically with ammonium ferrothiocyanate (Stewart 1980).

### Laser interferometric method

The amount of antibiotic (ampicillin or ciprofloxacin), *N*(*t*), which diffuses in time *t* from a liposomal solution to the water was calculated by integrating the concentration profile according to the formula

1 $${N(t) = {S}\int\limits_{0}^{\delta } {C_{1} (x,t)\text{d}x} }, $$

where *C*_1_(*x*,*t*) denotes the concentration of antibiotic at a point situated at a distance *x* from the gel–water interface, *S* is the surface area of the hydrophobic–hydrophilic interface (*S* = 7.0 × 10^−5^ m^2^), and *δ* is the concentration boundary layer (CBL) thickness.

The flux *J*_S_ of the antibiotic which flows through the hydrophobic–hydrophilic interface is given by

2 $$J_{\text{S}} = \frac{N(t)}{St}. $$

**Fig. 1:**
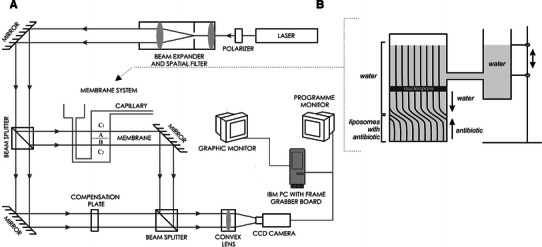
Scheme of the experimental setup (**a**) for interferometric investigations of substance transport and sketch of the measuring system (**b**)

The values *C*_1_(*x*, *t*) and *δ* were determined experimentally by means of laser interferometry. The scheme of the interferometric measurement system is presented in Fig. 1. It consists of a Mach–Zehnder interferometer with an He-Ne laser, two measurement cuvettes, a TV-CCD camera, and a computer with software for acquisition and processing of interference images (interferograms).

The interferograms, which appear due to the interference of two laser beams, are determined by the changes of refraction coefficient of the solute Δ*n*(*x*, *t*), affected by the concentration of the substance. When the solute is homogeneous, the interference fringes are straight, but they are bent when a concentration gradient appears. The concentration profile *C*(*x*, *t*) is determined by the deviation of the fringes from a straight course. Since the concentration *C* and the refraction coefficient are assumed to be linearly related, we have

3 $${C(x,t) = C_{0} + a{{\Updelta}}n(x,t) = C_{0} + a\frac{\lambda d(x,t)}{hf}}, $$

where *C*_0_ is the initial substance concentration, *a* is the proportionality constant between the concentration and the refraction index determined in a separate experiment using the interferometric refractometer (*a* = 11.59 × 10^3^ and 6.60 × 10^3^ mol/m^3^ for the ampicillin and ciprofloxacin aqueous solution, respectively), *λ* = 632.8 nm, the wavelength of the laser light, *h* is the distance between the fringes in the field where they are straight lines, and *f* is the thickness of the solution layer in the measurement cuvette. The CBL thickness *δ* was arbitrarily defined as the distance from the liposomal–water interface to the point at which the concentration decreases *k* times, i.e.,

4 $$C(t,x = 0) = kC(t,x = \delta ), $$

with *x* = 0 being the liposomal–water interface position. The arbitrary constant *k* is assumed to be equal to 12.5, but another value which satisfies specific application requirements may be adopted.

By recording interferograms at a given time interval, one can reconstruct the concentration profiles at different times. Such profiles were used to calculate the amount of transported antibiotic and concentration profile of ciprofloxacin or ampicillin as a function of distance from the liposomal–water interface. The image analysis software, among other things, allows one to ascertain the concentration profiles and CBL thicknesses. The interferograms are recorded from 0 to 2,400 s with time intervals of 120 s.

The measuring system consisted of two glass cuvettes (internal dimensions 10 × 7 × 70 mm) separated by horizontally located membrane. The polymeric track membrane used in the experiments exhibits a sufficiently high filtration coefficient that the water penetrated the membrane freely. By precise manipulation of the hydrostatic pressure control, the position of the water–solution free interface was controlled. The use of the membrane allowed us to avoid hydrodynamic instabilities during filling of the system. The upper cuvette was filled with pure water, while in the lower one neutral or anionic liposomal solution (1 mg/ml) with free antibiotic (1 mg/ml) was placed. The liposomal solutions at concentrations of 1 mg/ml without antibiotic were used as the control. All experiments were performed at temperature of 37 °C.

## Results and discussion

### Antibiotic diffusion from liposomal mixture

**Fig. 2:**
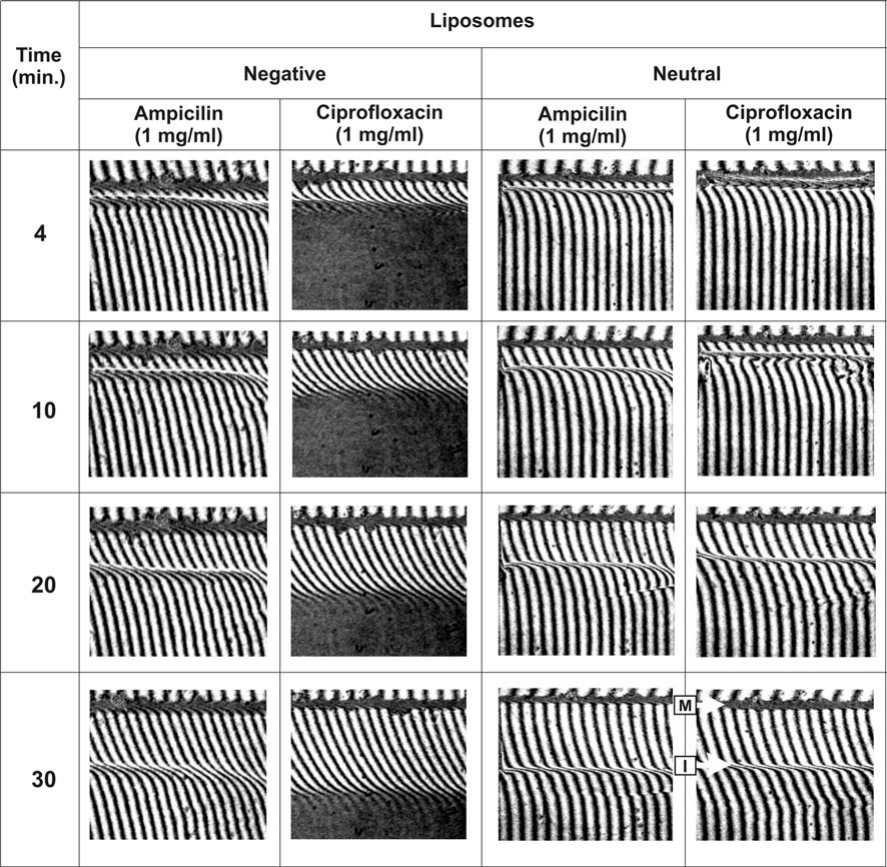
Interferometric images obtained at different times for ampicillin and ciprofloxacin released from anionic and neutral liposomal solutions. The arrows *M* and *I* indicate the membrane and the free water–liposomal solution interface, respectively

**Fig. 3:**
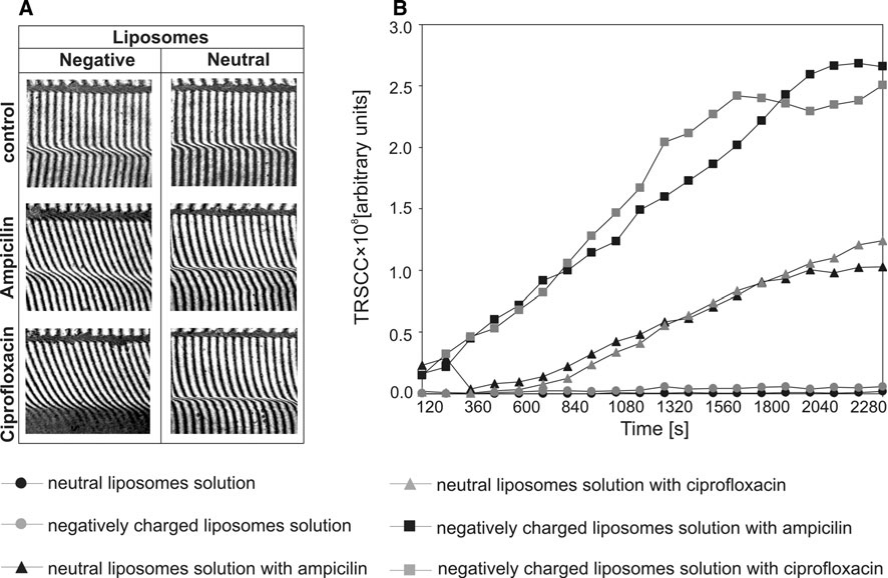
Comparison of interferograms (**a**) obtained after 40 min for liposomal solutions with antibiotic and control liposomal solutions (no antibiotic used). Total sum of refraction coefficient changes in the CBL region (**b**) obtained for solutions with antibiotic and control liposomal solutions

The interferograms taken at particular time intervals showed antibiotic diffusion from liposomal solution to the water phase (Fig. 2). The membrane (dark thick horizontal line) and free interface between liposomal solution and water (below the membrane) are visible. This interface is sharper for neutral liposomal solution, which indicates its higher hydrophobicity. The successive images show various stages of the CBL evolution. The thickness of the region where the fringes bend from a straight line represents the CBL thickness. Τhe thickness *δ* is relatively low (about 0.2 mm after 40 min) for control liposomal solutions (no antibiotic used), indicating that anionic and neutral liposomes practically do not diffuse into the water phase (Fig. 3a). According to the formula (4) for neutral liposomes–antibiotic mixture, the thicknesses *δ* were equal to 2.411 mm (ampicillin) and 2.391 mm (ciprofloxacin) after 40 min. For anionic lipid formulation mixed with antibiotic, these values were 2.353 and 2.1 mm for ampicillin and ciprofloxacin, respectively. It turned out that, in the case of liposome–antibiotic solution, only drug molecules diffuse into the water phase. The diffusion coefficient values for ampicillin and ciprofloxacin obtained from *δ* were about 4 × 10^−10^ m^2^/s, in agreement with literature data (Stewart 1996). The pattern of interferometric fringes also provided information about the antibiotic distribution within the measured probe. The right bending of the fringes from the straight line (above the water–liposomal solution interface) reflected an increase of antibiotic concentration in the water compartment, while the left bending of the fringes below the water–liposomal solution interface corresponded to a decrease of antibiotic in the CBL. It was observed that the amount of water which permeated into the liposomal–antibiotic solution was higher than the amount of water penetrating the solution with no antibiotic (Fig. 3a). In Fig. 3a, a comparison of interferograms obtained after 40 min for control liposomal solutions (without the antibiotic) and liposome–antibiotic solutions is shown. It might suggest a decrease of liposomal solution hydrophobicity in the presence of antibiotics.

As mentioned above, the basis of interferometric diffusion analysis is the change of the refraction coefficient of the solute Δ*n*(*x*, *t*). The total sum of refraction coefficient changes (TSRCC) in the CBL region is calculated by the formula

5 $${{\text{TSRCC}}(t) = {{S}}\int\limits_{0}^{\delta } {{{\Updelta}}n(x,t)\text{d}x} }. $$

**Fig. 4:**
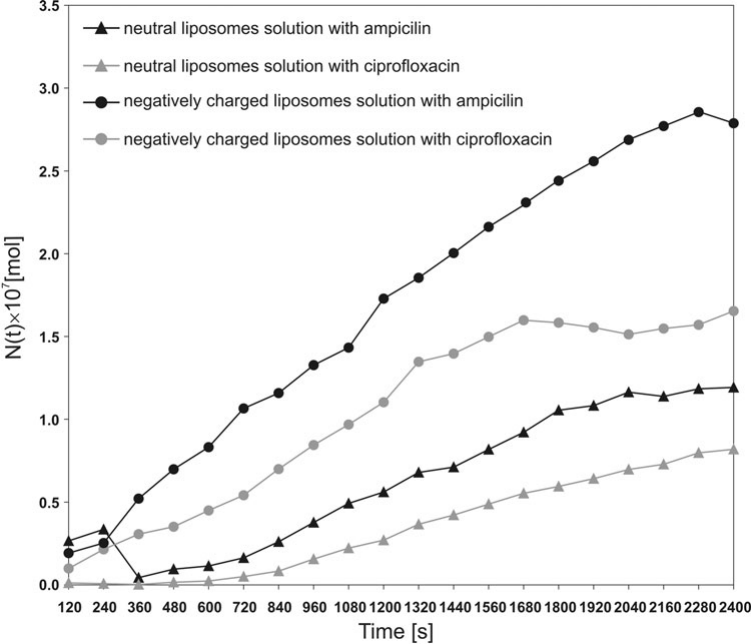
Time dependencies of the amount of ampicillin and ciprofloxacin released to the water from the anionic and neutral liposomal solutions

It reflects the amount of substance contained in the CBL region after time *t*, shown in Fig. 3b. The release from control liposomal solutions was very small in comparison with the liposomal solutions with antibiotic; For example, after 40 min, the amount of substance released from control solution (lipid diffusion) reached about 2 % of the total amount of substance released from solution with antibiotic. Additionally, the anionic liposomal formulation was less hydrophobic than neutral ones, because the amount of liposomes released from anionic control solution was about two times higher. The diffusion of anionic and neutral liposomes into the water was negligible, and during the experiment practically only the diffusion of pure antibiotic was measured. To calculate the amount of transported antibiotic, *N*(*t*), the TSRCC(*t*) values should be multiplied by the coefficient *a* mentioned above. The time dependencies *N*(*t*) of the amount of antibiotic transported from the liposomal solution to the water phase increased in a nonlinear manner (Fig. 4). The diffusion kinetics of antibiotics released from neutral and negatively charged liposome solutions differed. For the initial time (0–600 s), the amounts of antibiotics released from the neutral solution were very small. Then the amount of transported substance increased uniformly, and after 2,000 s a decrease of transported substance (particularly for ampicillin) was noted. For negatively charged liposomes the amount of transported substance increased rapidly until the “plateau effect” was reached, described as the system steady state, for ciprofloxacin and ampicillin after 1,600 and 2,300 s, respectively. Comparison of the amount of antibiotics transported from both tested solutions indicates that negative liposomal formulation mixtures release drugs more easily than neutral ones. The ampicillin was released more efficiently than ciprofloxacin regardless of the liposomal solution type. In general, after 40 min, about 2.40 × 10^−7^ and 1.65 × 10^−7^ mol of ampicillin and ciprofloxacin were released from solution containing negatively charged liposomes, respectively. At the same time only 1.19 × 10^−7^ mol of ampicillin and 8.19 × 10^−8^ mol of ciprofloxacin diffused from neutral liposome solution.

**Fig. 5:**
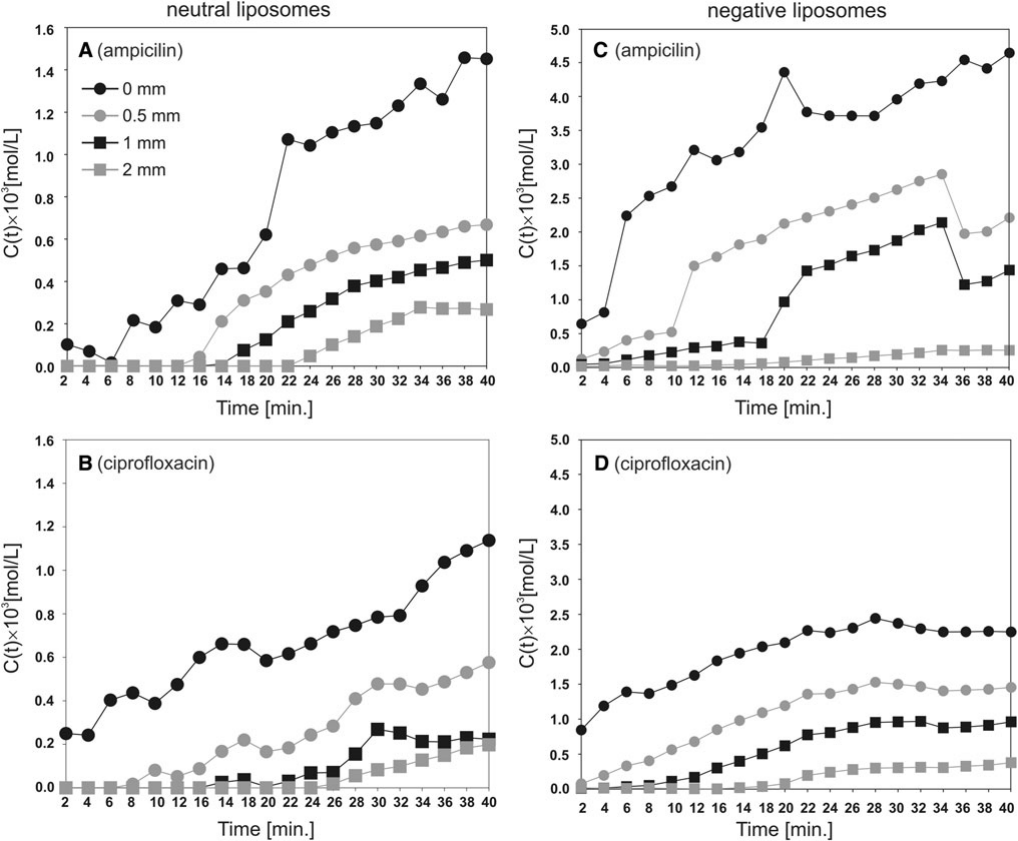
Concentration field evolution for ampicillin (**a**) and ciprofloxacin (**b**) released from neutral liposomal solution and for ampicillin (**c**) and ciprofloxacin, (**d**) released from negatively charged liposomal solution (detailed description in text)

Figure 5a–d shows the time characteristics of the antibiotic concentration at selected points, at distances *x*_0_ = 0, 0.5, 1, and 2 mm from the water–solution interface. Curve analysis provided essential information about the concentration field evolution in the tested samples. In all cases the highest concentration occurred at the water–solution interface (*x*_0_ = 0 mm). A concentration of zero indicates that we are outside the concentration boundary region; for example, the diffusing ampicillin reached the point *x*_0_ = 2 mm after time *t* > 1,300 s (Fig. 5a). At the same time, at points *x*_0_ = 1, 0.5, and 0 mm, the concentrations reached the values of 2.1 × 10^−4^, 4.31 × 10^−4^, and 1.07 × 10^−3^ mol/m^3^, respectively. Analysis of the presented dependencies indicated that the concentration field evolution at greater distances from the water–liposomal solution interface (*x*_0_ = 1 and 2 mm) was similar for both investigated solutions and antibiotics. The concentration increased practically proportionally in a time-dependent manner, and the dynamics of these changes were small. At the points *x*_0_ = 0 and 0.5 mm, the dynamics of the concentration changes of the investigated antibiotics were different for neutral and negatively charged liposomes. For neutral solution (Fig. 5a, b) the concentration also increased proportionally with time. However, for anionic solution (Fig. 5c, d) high concentration increases for times 0–800 s were observed whereas for longer times the concentration changes were detected near to zero. Such a phenomenon was observed for ampicillin as well as for ciprofloxacin.

**Fig. 6:**
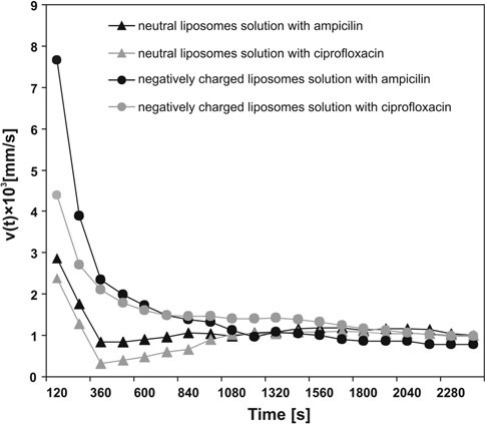
Time characteristics of expansion velocity of ampicillin and ciprofloxacin released from neutral and anionic liposomal solutions

The velocity of the concentration boundary layer’s “growth” *v* = *δ*/*t*, which refers to the concentration field velocity in the system, is presented in Fig. 6. The CBLs that are created near to the edge of phases cause the concentration polarization of the system and strongly affect the transport processes in artificial (Dworecki and Wąsik 1997; Dworecki et al. 2000, 2003, 2005a, b, 2006; Ślęzak et al. 2005; Wąsik et al. 2010; Murphy et al. 1992; Zabolotsky et al. 1996, 2002; Sistat and Pourcelly 1997; Pohl et al. 1998; Rubinstein and Zaltzman 2000; Zydney 1997; Pismenskaya et al. 2001; Larchet et al. 2008; Shaposhnik et al. 2008; Nikonenko et al. 2009; Kozmai et al. 2010) as well as biological systems (House 1974; Barry and Diamond 1984; Winne 1981; Levitt et al. 1992; Fischbarg et al. 1993; Cotton and Reuss 1989).

The course of the time characteristic *v*(*t*) for both investigated antibiotics is similar; however, higher values of *v* were observed for ampicillin regardless of the liposomal solution type. The value of *v*(*t*) decreased nonmonotonically with time. For initial times the decrease was particularly rapid. This was caused by the concentration polarization of the system. For times *t* > 1,000 s we observed a very slow decrease of *v* (practically constant). In the range 0–800 s, the values of *v* for both released antibiotics were higher for anionic than for neutral solution; For example, at the time 120 s, we obtained 7.7 × 10^−3^ for ampicillin and 4.4 × 10^−3^ mm/s for ciprofloxacin released from anionic solution. If antibiotics were released from neutral solution, these values were 2.9 × 10^−3^ mm/s and 2.4 × 10^−3^ mm/s, respectively. For longer times of *t* > 1,000 s, the values of *v* were practically identical (1 × 10^−3^ mm/s) for both investigated antibiotics regardless of the solution type.

As seen, the differences occurred for initial times, which means that they were caused not by diffusion in the water phase but probably by different mechanisms of release of antibiotics from neutral and anionic solutions. Moreover, the noticeable high value of *v* for ampicillin released from anionic solution showed a strong interaction between this substance and negatively charged liposomal solution. Practically identical values of *v* for long times for antibiotics released from neutral as well as from anionic solutions indicate that the transport kinetics of these substances in the water phase were similar.

### Antibiotic–liposome interactions

Based on the analysis of interferograms (Fig. 2), it could be observed that anionic liposomal solution with ciprofloxacin strongly absorbed red laser light. This indicates that ciprofloxacin disrupted the anionic vesicles. Ciprofloxacin is an antibacterial agent of the 4-quinolone group derived by systematic modification of nalidixic acid. The antibacterial properties of ciprofloxacin are associated with (a) inhibition of intracellular enzymes (DNA-gyrase, topoisomerase IV) and (b) the efficient transport of antibiotic molecules through bacterial envelopes and cytoplasmic membranes (Weigel et al. 1998; Vila et al. 1997; Hernández-Borrell and Teresa Montero 2003). It is suggested that this second property of ciprofloxacin is related to a broad spectrum of action against Gram-positive as well as Gram-negative bacteria. The physicochemical properties of ciprofloxacin (an amphoteric molecule with two potential ionizable groups) facilitate its interaction with lipids in the membrane structure. Ciprofloxacin formed four different microspecies (neutral, zwitterion, and positively and negatively charged) depending on the pH of the solution (Hernández-Borrell and Teresa Montero 2003). Our results confirm that ciprofloxacin at neutral pH shows little tendency to bind neutral liposomes (Maurer et al. 1998). Additionally we observed by the laser interferometry method that ciprofloxacin disrupts negatively charged liposomes as a final effect of antibiotic–lipid interactions. We concluded that the positively charged piperazine ring at the C-7 position of ciprofloxacin might interact with negatively charged headgroups of phospholipids (Hernández-Borrell and Teresa Montero 2003; Montero et al. 1996; Bensikaddour et al. 2008). In contrast to ciprofloxacin, ampicillin like β-lactam does not change the internal osmotic pressure or the optical density of liposomes.

In conclusion, an optimized laser interferometry system without an artificial boundary of phases was used for analysis of the liposome and antibiotic diffusion process. The diffusion of anionic and neutral liposomes into the water phase was insignificant, and mainly the diffusion of antibiotics was measured. Differences in the diffusion kinetics of both antibiotics from liposomal solution to the water phase were observed. Moreover, the amount of ampicillin and ciprofloxacin released from the anionic liposomal phase was higher than that from the neutral one. The novel laser interferometry system turned out to be a very useful tool for quantitative analysis of the lipid and antibiotic diffusion process from a hydrophobic mixture to the water phase.
